# Correction: Thermodynamically stable vesicle formation of biodegradable double mPEG-tailed amphiphiles with sulfonate head group

**DOI:** 10.1039/d0ra90114h

**Published:** 2020-11-02

**Authors:** Rita Ghosh, Joykrishna Dey, B. V. N. Phani Kumar

**Affiliations:** Department of Chemistry, Indian Institute of Technology Kharagpur Kharagpur-721302 India joydey@chem.iitkgp.ac.in +91-3222-255303 +91-3222-283308; NMR, CATERS, CSIR-Central Leather Research Institute Adyar Chennai-600020 India

## Abstract

Correction for ‘Thermodynamically stable vesicle formation of biodegradable double mPEG-tailed amphiphiles with sulfonate head group’ by Rita Ghosh *et al.*, *RSC Adv.*, 2020, **10**, 32522–32531, DOI: 10.1039/D0RA05613H

The authors regret that an incorrect version of Fig. 5 was included in the original article. The correct version of Fig. 5 is presented below.

**Fig. 1:**
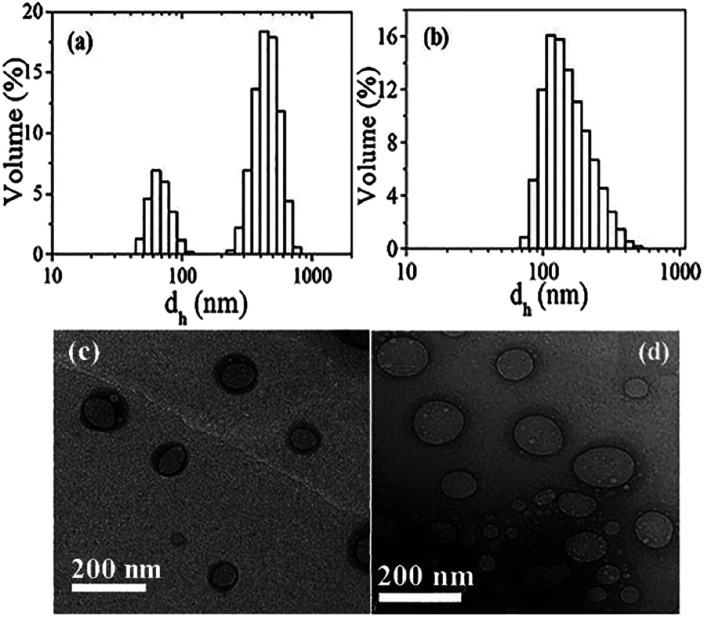
Upper panel: Size distribution histograms of aggregates in 2.0 mM solutions (pH 7) of (a) (mPEG_4_)_2_SO_3_Na, and (b) (mPEG_23_)_2_SO_3_Na at 25 °C; lower panel: unstained HRTEM images of 2 mM (c) (mPEG_4_)_2_SO_3_Na and (d) (mPEG_23_)_2_SO_3_Na solutions in phosphate buffer (pH 7.0).

The Royal Society of Chemistry apologises for these errors and any consequent inconvenience to authors and readers.

## Supplementary Material

